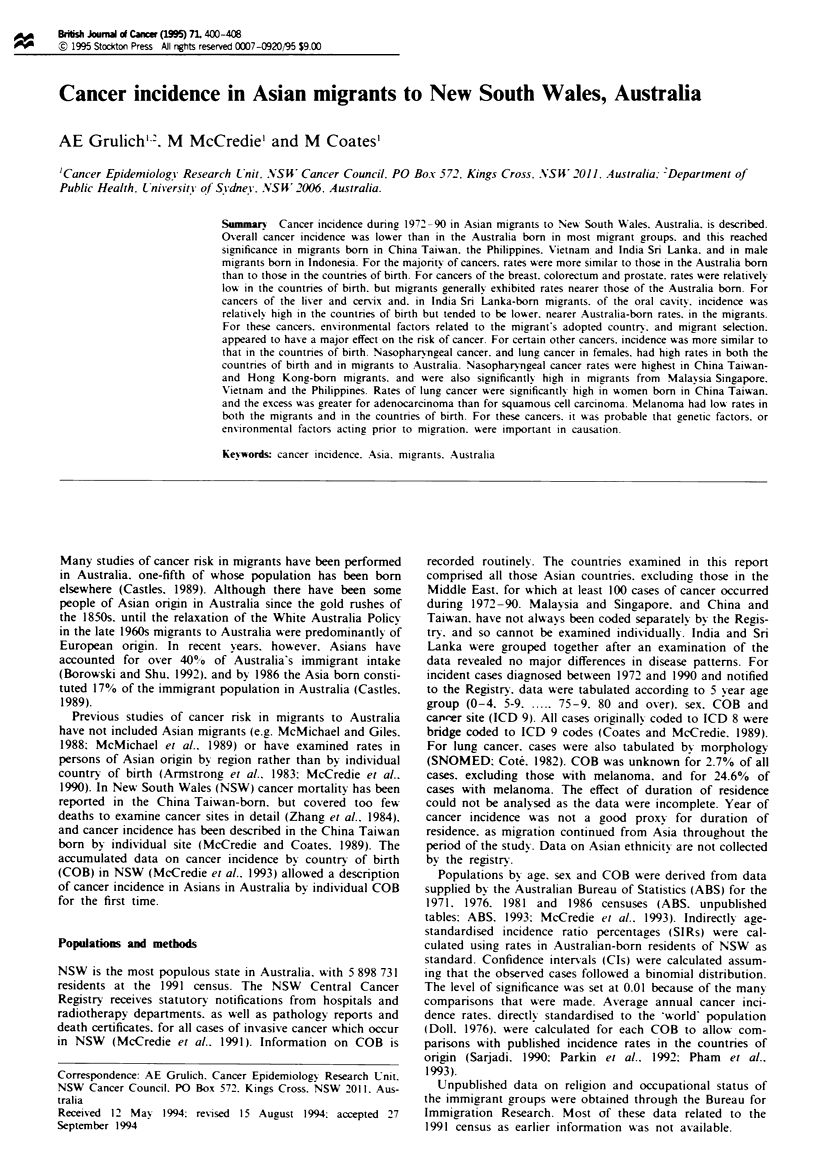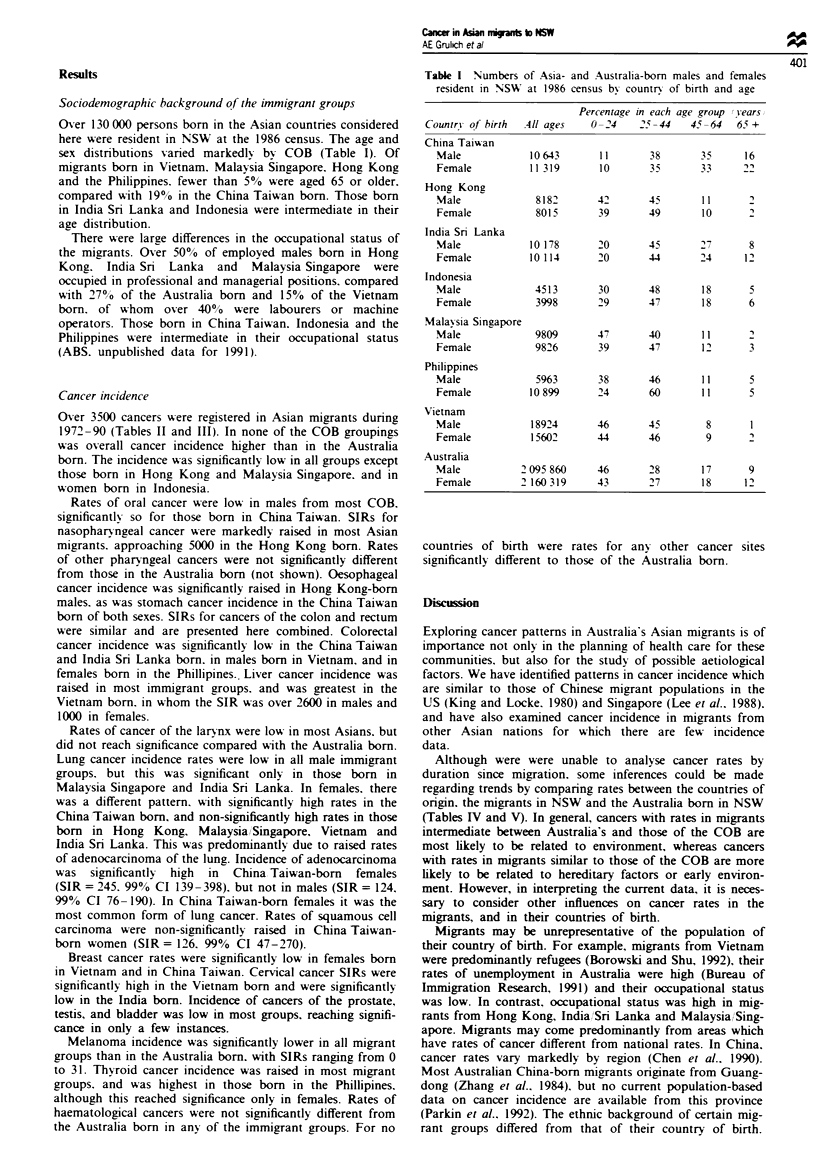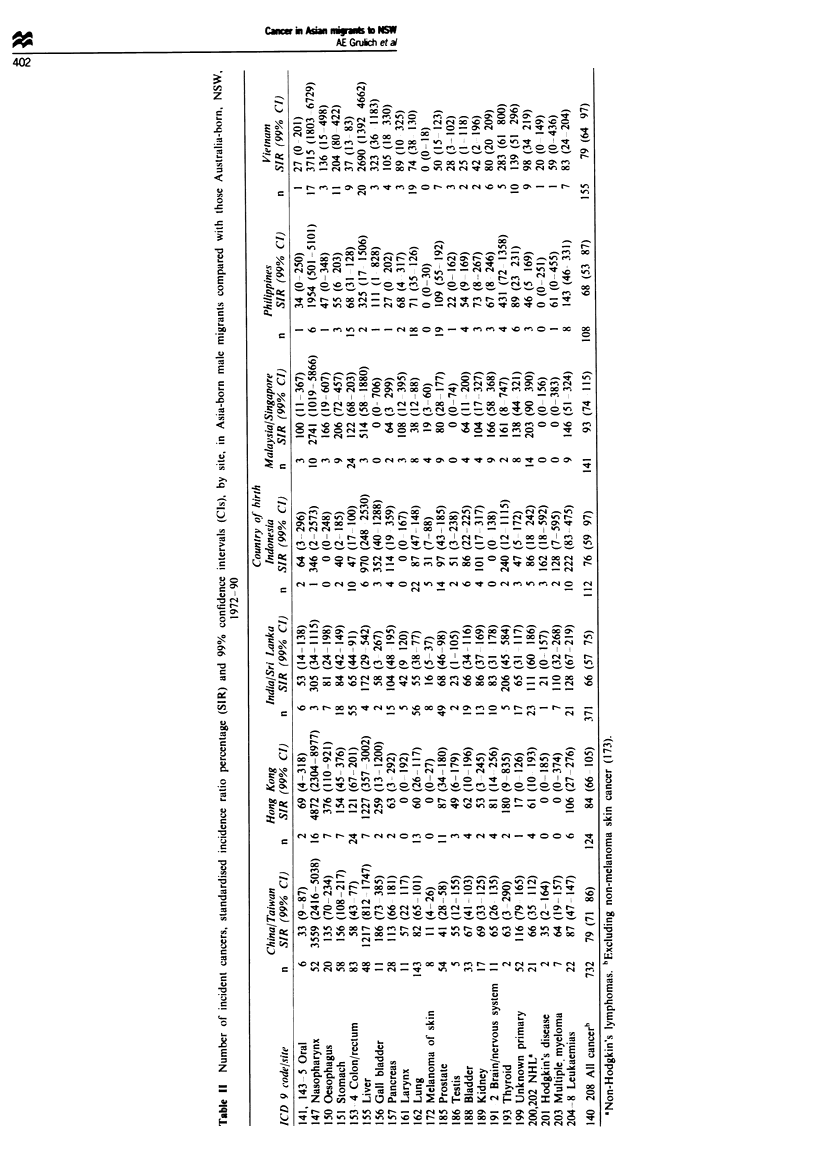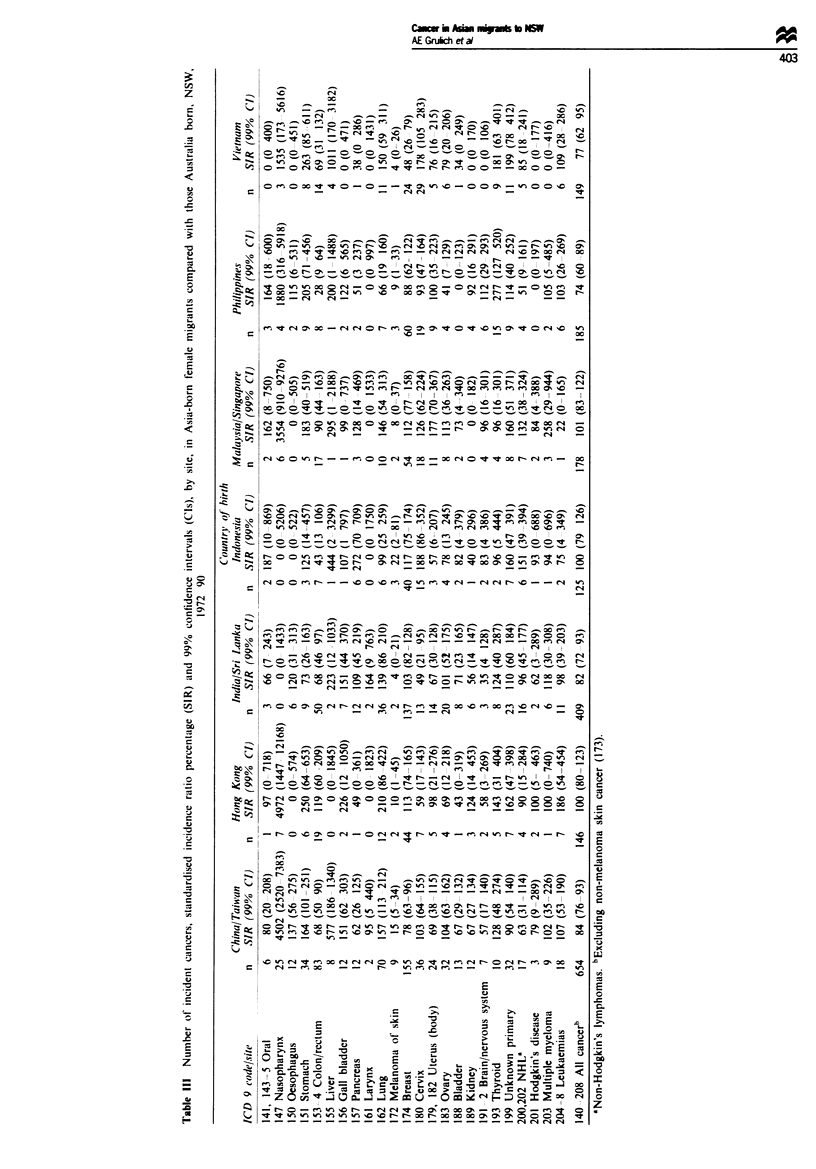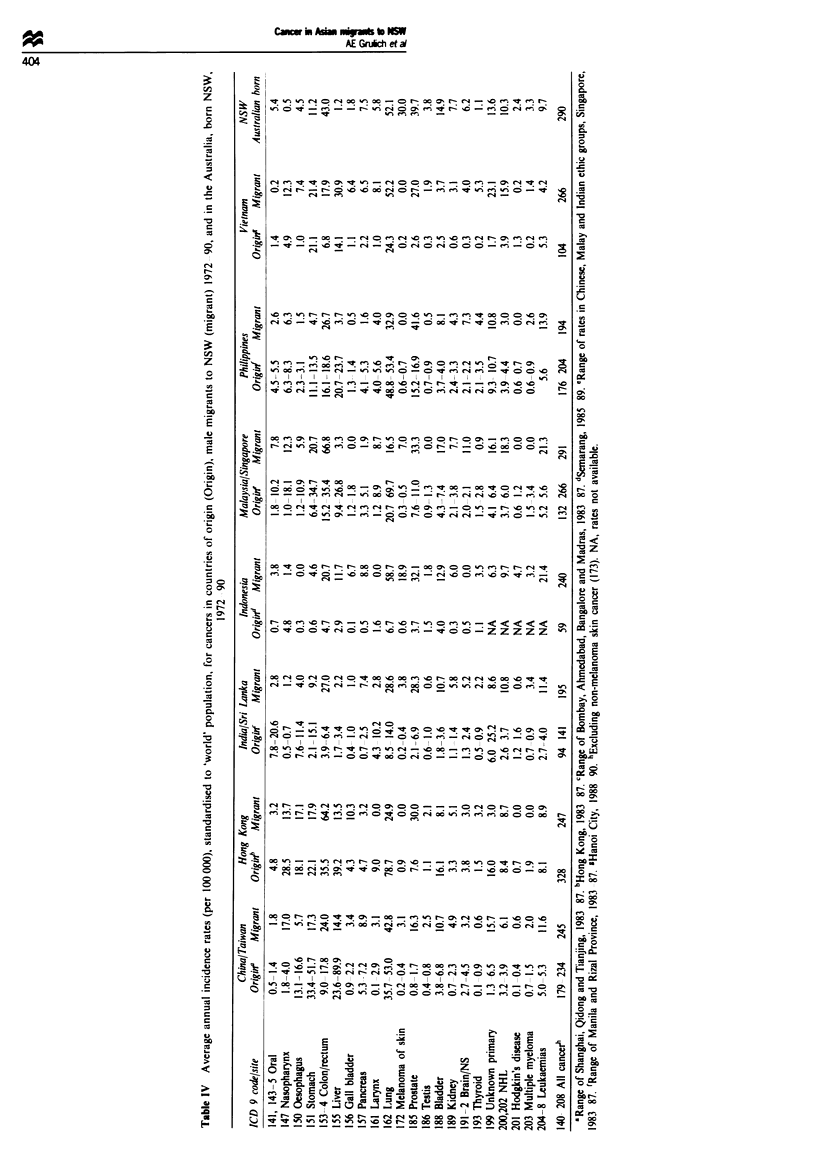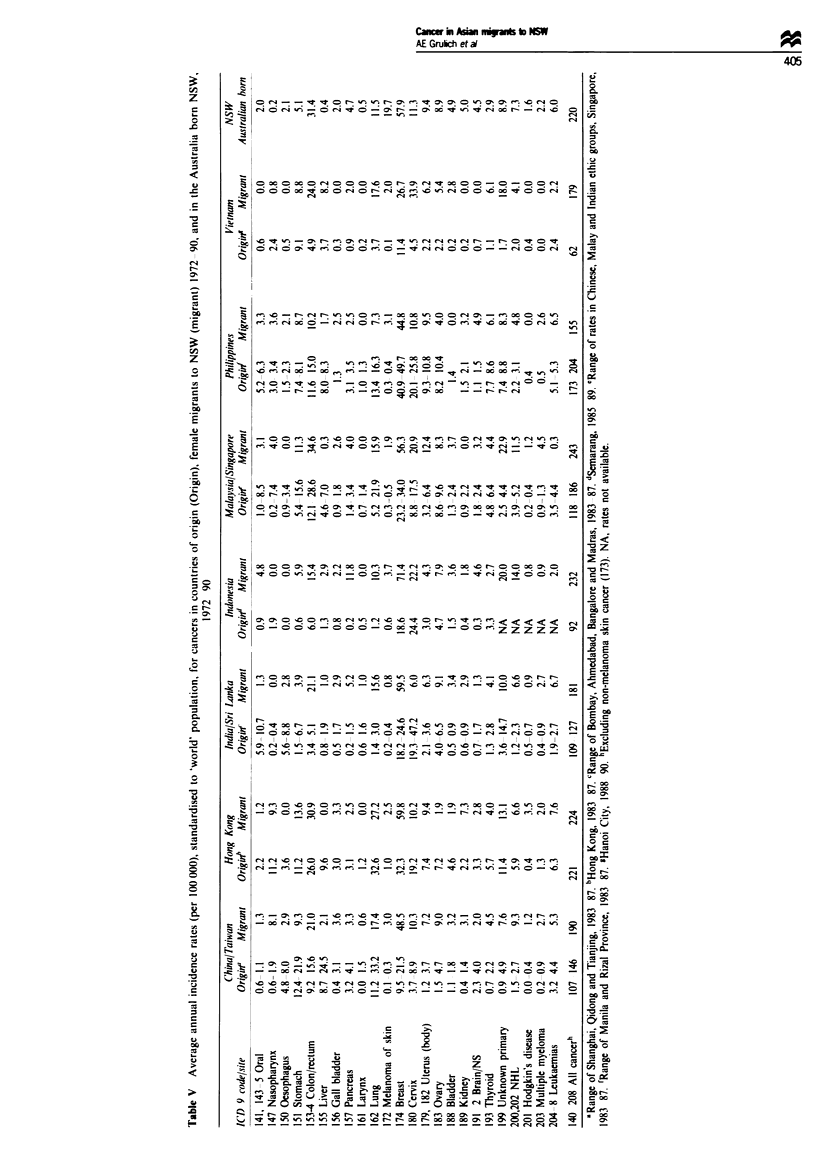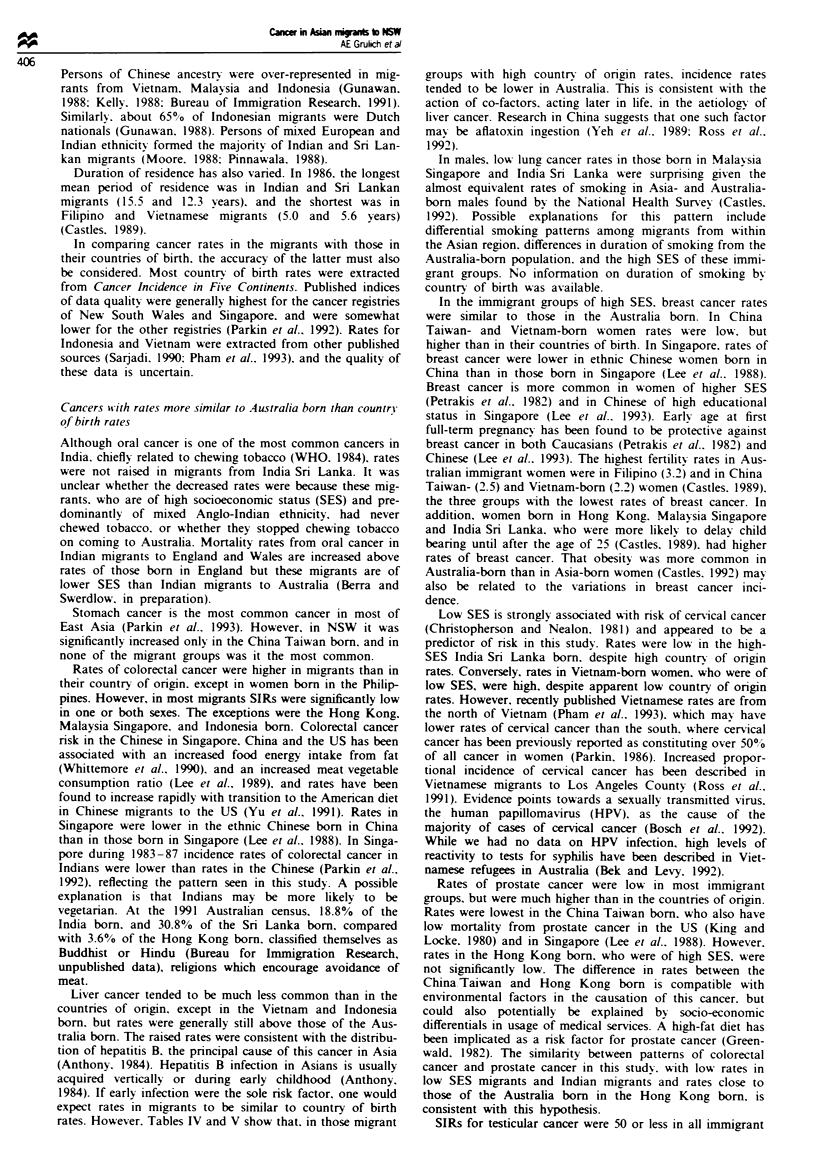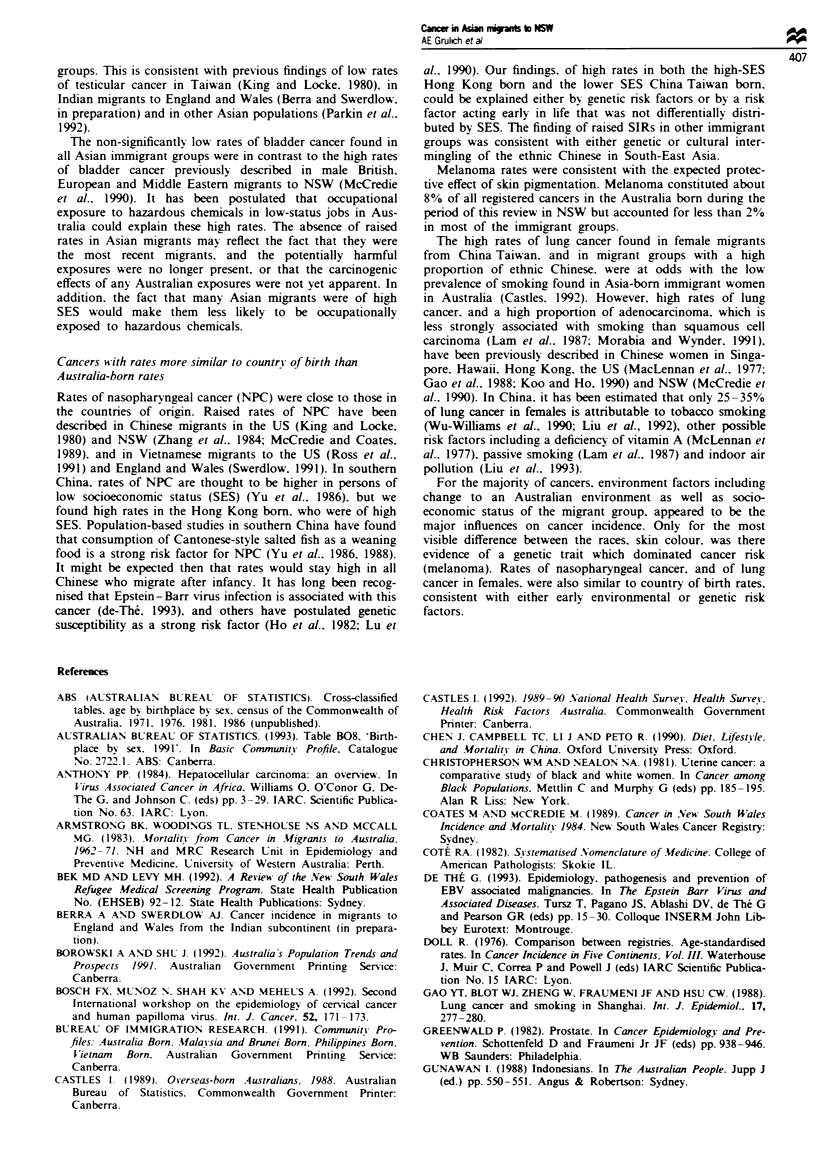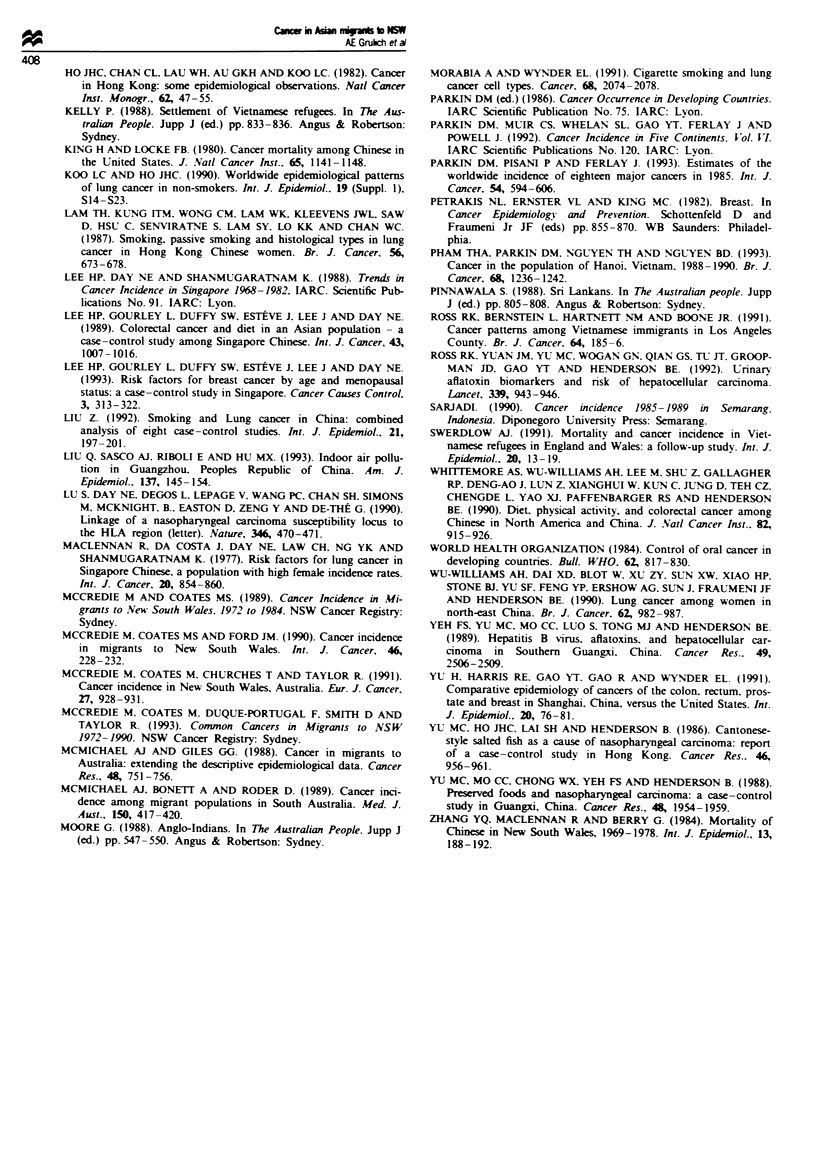# Cancer incidence in Asian migrants to New South Wales, Australia.

**DOI:** 10.1038/bjc.1995.82

**Published:** 1995-02

**Authors:** A. E. Grulich, M. McCredie, M. Coates

**Affiliations:** Cancer Epidemiology Research Unit, NSW Cancer Council, Australia.

## Abstract

Cancer incidence during 1972-90 in Asian migrants to New South Wales, Australia, is described. Overall cancer incidence was lower than in the Australia born in most migrant groups, and this reached significance in migrants born in China/Taiwan, the Philippines, Vietnam and India/Sri Lanka, and in male migrants born in Indonesia. For the majority of cancers, rates were more similar to those in the Australia born than to those in the countries of birth. For cancers of the breast, colorectum and prostate, rates were relatively low in the countries of birth, but migrants generally exhibited rates nearer those of the Australia born. For cancers of the liver and cervix and, in India/Sri Lanka-born migrants, of the oral cavity, incidence was relatively high in the countries of birth but tended to be lower, nearer Australia-born rates, in the migrants. For these cancers, environmental factors related to the migrant's adopted country, and migrant selection, appeared to have a major effect on the risk of cancer. For certain other cancers, incidence was more similar to that in the countries of birth. Nasopharyngeal cancer, and lung cancer in females, had high rates in both the countries of birth and in migrants to Australia. Nasopharyngeal cancer rates were highest in China/Taiwan and Hong Kong-born migrants, and were also significantly high in migrants from Malaysia/Singapore, Vietnam and the Philippines. Rates of lung cancer were significantly high in women born in China/Taiwan, and the excess was greater for adenocarcinoma than for squamous cell carcinoma. Melanoma had low rates in both the migrants and in the countries of birth. For these cancers, it was probable that genetic factors, or environmental factors acting prior to migration, were important in causation.


British Journal of Cancer (1995) 71, 400-408

© 1995 Stockton Press All rights reserved 0007-0920/95 $9.00

### Cancer incidence in Asian migrants to New South Wales, Australia

AE Grulich[1,2], M McCredie[1] and M Coates[1]

[1]*Cancer Epidemiology Research Unit, NSW Cancer Council, PO Box 572, Kings Cross, NSW 2011, Australia;* [2]*Department of Public Health, University of Sydney, NSW 2006, Australia.*

**Summary** Cancer incidence during 1972-90 in Asian migrants to New South Wales, Australia, is described. Overall cancer incidence was lower than in the Australia born in most migrant groups, and this reached significance in migrants born in China Taiwan, the Philippines, Vietnam and India Sri Lanka, and in male migrants born in Indonesia. For the majority of cancers, rates were more similar to those in the Australia born than to those in the countries of birth. For cancers of the breast, colorectum and prostate, rates were relatively low in the countries of birth, but migrants generally exhibited rates nearer those of the Australia born. For cancers of the liver and cervix and, in India Sri Lanka-born migrants, of the oral cavity, incidence was relatively high in the countries of birth but tended to be lower, nearer Australia-born rates, in the migrants. For these cancers, environmental factors related to the migrant's adopted country, and migrant selection, appeared to have a major effect on the risk of cancer. For certain other cancers, incidence was more similar to that in the countries of birth. Nasopharyngeal cancer, and lung cancer in females, had high rates in both the countries of birth and in migrants to Australia. Nasopharyngeal cancer rates were highest in China Taiwan- and Hong Kong-born migrants, and were also significantly high in migrants from Malaysia Singapore, Vietnam and the Philippines. Rates of lung cancer were significantly high in women born in China Taiwan, and the excess was greater for adenocarcinoma than for squamous cell carcinoma. Melanoma had low rates in both the migrants and in the countries of birth. For these cancers, it was probable that genetic factors, or environmental factors acting prior to migration, were important in causation.

**Keywords:** cancer incidence, Asia, migrants, Australia

Many studies of cancer risk in migrants have been performed in Australia, one-fifth of whose population has been born elsewhere (Castles, 1989). Although there have been some people of Asian origin in Australia since the gold rushes of the 1850s, until the relaxation of the White Australia Policy in the late 1960s migrants to Australia were predominantly of European origin. In recent years, however, Asians have accounted for over 40% of Australia's immigrant intake (Borowski and Shu, 1992), and by 1986 the Asia born constituted 17% of the immigrant population in Australia (Castles, 1989).

Previous studies of cancer risk in migrants to Australia have not included Asian migrants (e.g. McMichael and Giles, 1988; McMichael *et al.*, 1989) or have examined rates in persons of Asian origin by region rather than by individual country of birth (Armstrong *et al.*, 1983; McCredie *et al.*, 1990). In New South Wales (NSW) cancer mortality has been reported in the China Taiwan-born, but covered too few deaths to examine cancer sites in detail (Zhang *et al.*, 1984), and cancer incidence has been described in the China Taiwan born by individual site (McCredie and Coates, 1989). The accumulated data on cancer incidence by country of birth (COB) in NSW (McCredie *et al.*, 1993) allowed a description of cancer incidence in Asians in Australia by individual COB for the first time.

### Populations and methods

NSW is the most populous state in Australia, with 5 898 731 residents at the 1991 census. The NSW Central Cancer Registry receives statutory notifications from hospitals and radiotherapy departments, as well as pathology reports and death certificates, for all cases of invasive cancer which occur in NSW (McCredie *et al.*, 1991). Information on COB is recorded routinely. The countries examined in this report comprised all those Asian countries, excluding those in the Middle East, for which at least 100 cases of cancer occurred during 1972-90. Malaysia and Singapore, and China and Taiwan, have not always been coded separately by the Registry, and so cannot be examined individually. India and Sri Lanka were grouped together after an examination of the data revealed no major differences in disease patterns. For incident cases diagnosed between 1972 and 1990 and notified to the Registry, data were tabulated according to 5 year age group (0-4, 5-9, ....., 75-9, 80 and over), sex, COB and cancer site (ICD 9). All cases originally coded to ICD 8 were bridge coded to ICD 9 codes (Coates and McCredie, 1989). For lung cancer, cases were also tabulated by morphology (SNOMED; Coté, 1982). COB was unknown for 2.7% of all cases, excluding those with melanoma, and for 24.6% of cases with melanoma. The effect of duration of residence could not be analysed as the data were incomplete. Year of cancer incidence was not a good proxy for duration of residence, as migration continued from Asia throughout the period of the study. Data on Asian ethnicity are not collected by the registry.

Populations by age, sex and COB were derived from data supplied by the Australian Bureau of Statistics (ABS) for the 1971, 1976, 1981 and 1986 censuses (ABS, unpublished tables; ABS, 1993; McCredie *et al.*, 1993). Indirectly age-standardised incidence ratio percentages (SIRs) were calculated using rates in Australian-born residents of NSW as standard. Confidence intervals (CIs) were calculated assuming that the observed cases followed a binomial distribution. The level of significance was set at 0.01 because of the many comparisons that were made. Average annual cancer incidence rates, directly standardised to the 'world' population (Doll, 1976), were calculated for each COB to allow comparisons with published incidence rates in the countries of origin (Sarjadi, 1990; Parkin *et al.*, 1992; Pham *et al.*, 1993).

Unpublished data on religion and occupational status of the immigrant groups were obtained through the Bureau for Immigration Research. Most of these data related to the 1991 census as earlier information was not available.

Correspondence: AE Grulich, Cancer Epidemiology Research Unit, NSW Cancer Council, PO Box 572, Kings Cross, NSW 2011, Australia
Received 12 May 1994; revised 15 August 1994; accepted 27 September 1994

**Results**

*Sociodemographic background of the immigrant groups*

Over 130 000 persons born in the Asian countries considered here were resident in NSW at the 1986 census. The age and sex distributions varied markedly by COB (Table I). Of migrants born in Vietnam, Malaysia Singapore, Hong Kong and the Philippines, fewer than 5% were aged 65 or older, compared with 19% in the China Taiwan born. Those born in India Sri Lanka and Indonesia were intermediate in their age distribution.

There were large differences in the occupational status of the migrants. Over 50% of employed males born in Hong Kong, India Sri Lanka and Malaysia Singapore were occupied in professional and managerial positions, compared with 27% of the Australia born and 15% of the Vietnam born, of whom over 40% were labourers or machine operators. Those born in China Taiwan, Indonesia and the Philippines were intermediate in their occupational status (ABS, unpublished data for 1991).

*Cancer incidence*

Over 3500 cancers were registered in Asian migrants during 1972–90 (Tables II and III). In none of the COB groupings was overall cancer incidence higher than in the Australia born. The incidence was significantly low in all groups except those born in Hong Kong and Malaysia Singapore, and in women born in Indonesia.

Rates of oral cancer were low in males from most COB, significantly so for those born in China Taiwan. SIRs for nasopharyngeal cancer were markedly raised in most Asian migrants, approaching 5000 in the Hong Kong born. Rates of other pharyngeal cancers were not significantly different from those in the Australia born (not shown). Oesophageal cancer incidence was significantly raised in Hong Kong-born males, as was stomach cancer incidence in the China Taiwan born of both sexes. SIRs for cancers of the colon and rectum were similar and are presented here combined. Colorectal cancer incidence was significantly low in the China Taiwan and India Sri Lanka born, in males born in Vietnam, and in females born in the Phillipines. Liver cancer incidence was raised in most immigrant groups, and was greatest in the Vietnam born, in whom the SIR was over 2600 in males and 1000 in females.

Rates of cancer of the larynx were low in most Asians, but did not reach significance compared with the Australia born. Lung cancer incidence rates were low in all male immigrant groups, but this was significant only in those born in Malaysia Singapore and India Sri Lanka. In females, there was a different pattern, with significantly high rates in the China Taiwan born, and non-significantly high rates in those born in Hong Kong, Malaysia/Singapore, Vietnam and India Sri Lanka. This was predominantly due to raised rates of adenocarcinoma of the lung. Incidence of adenocarcinoma was significantly high in China Taiwan-born females (SIR = 245, 99% CI 139–398), but not in males (SIR = 124, 99% CI 76–190). In China Taiwan-born females it was the most common form of lung cancer. Rates of squamous cell carcinoma were non-significantly raised in China Taiwan-born women (SIR = 126, 99% CI 47–270).

Breast cancer rates were significantly low in females born in Vietnam and in China Taiwan. Cervical cancer SIRs were significantly high in the Vietnam born and were significantly low in the India born. Incidence of cancers of the prostate, testis, and bladder was low in most groups, reaching significance in only a few instances.

Melanoma incidence was significantly lower in all migrant groups than in the Australia born, with SIRs ranging from 0 to 31. Thyroid cancer incidence was raised in most migrant groups, and was highest in those born in the Phillipines, although this reached significance only in females. Rates of haematological cancers were not significantly different from the Australia born in any of the immigrant groups. For no countries of birth were rates for any other cancer sites significantly different to those of the Australia born.

**Table I** Numbers of Asia- and Australia-born males and females resident in NSW at 1986 census by country of birth and age

| *Country of birth* | *All ages* | *Percentage in each age group (years)* 0–24 | 25–44 | 45–64 | 65 + |
|---|---|---|---|---|---|
| China Taiwan | | | | | |
| Male | 10 643 | 11 | 38 | 35 | 16 |
| Female | 11 319 | 10 | 35 | 33 | 22 |
| Hong Kong | | | | | |
| Male | 8182 | 42 | 45 | 11 | 2 |
| Female | 8015 | 39 | 49 | 10 | 2 |
| India Sri Lanka | | | | | |
| Male | 10 178 | 20 | 45 | 27 | 8 |
| Female | 10 114 | 20 | 44 | 24 | 12 |
| Indonesia | | | | | |
| Male | 4513 | 30 | 48 | 18 | 5 |
| Female | 3998 | 29 | 47 | 18 | 6 |
| Malaysia Singapore | | | | | |
| Male | 9809 | 47 | 40 | 11 | 2 |
| Female | 9826 | 39 | 47 | 12 | 3 |
| Philippines | | | | | |
| Male | 5963 | 38 | 46 | 11 | 5 |
| Female | 10 899 | 24 | 60 | 11 | 5 |
| Vietnam | | | | | |
| Male | 18924 | 46 | 45 | 8 | 1 |
| Female | 15602 | 44 | 46 | 9 | 2 |
| Australia | | | | | |
| Male | 2 095 860 | 46 | 28 | 17 | 9 |
| Female | 2 160 319 | 43 | 27 | 18 | 12 |

**Discussion**

Exploring cancer patterns in Australia's Asian migrants is of importance not only in the planning of health care for these communities, but also for the study of possible aetiological factors. We have identified patterns in cancer incidence which are similar to those of Chinese migrant populations in the US (King and Locke, 1980) and Singapore (Lee *et al.*, 1988), and have also examined cancer incidence in migrants from other Asian nations for which there are few incidence data.

Although were were unable to analyse cancer rates by duration since migration, some inferences could be made regarding trends by comparing rates between the countries of origin, the migrants in NSW and the Australia born in NSW (Tables IV and V). In general, cancers with rates in migrants intermediate between Australia's and those of the COB are most likely to be related to environment, whereas cancers with rates in migrants similar to those of the COB are more likely to be related to hereditary factors or early environment. However, in interpreting the current data, it is necessary to consider other influences on cancer rates in the migrants, and in their countries of birth.

Migrants may be unrepresentative of the population of their country of birth. For example, migrants from Vietnam were predominantly refugees (Borowski and Shu, 1992), their rates of unemployment in Australia were high (Bureau of Immigration Research, 1991) and their occupational status was low. In contrast, occupational status was high in migrants from Hong Kong, India/Sri Lanka and Malaysia/Singapore. Migrants may come predominantly from areas which have rates of cancer different from national rates. In China, cancer rates vary markedly by region (Chen *et al.*, 1990). Most Australian China-born migrants originate from Guangdong (Zhang *et al.*, 1984), but no current population-based data on cancer incidence are available from this province (Parkin *et al.*, 1992). The ethnic background of certain migrant groups differed from that of their country of birth.

Persons of Chinese ancestry were over-represented in migrants from Vietnam, Malaysia and Indonesia (Gunawan, 1988; Kelly, 1988; Bureau of Immigration Research, 1991). Similarly, about 65% of Indonesian migrants were Dutch nationals (Gunawan, 1988). Persons of mixed European and Indian ethnicity formed the majority of Indian and Sri Lankan migrants (Moore, 1988; Pinnawala, 1988).

Duration of residence has also varied. In 1986, the longest mean period of residence was in Indian and Sri Lankan migrants (15.5 and 12.3 years), and the shortest was in Filipino and Vietnamese migrants (5.0 and 5.6 years) (Castles, 1989).

In comparing cancer rates in the migrants with those in their countries of birth, the accuracy of the latter must also be considered. Most country of birth rates were extracted from *Cancer Incidence in Five Continents*. Published indices of data quality were generally highest for the cancer registries of New South Wales and Singapore, and were somewhat lower for the other registries (Parkin *et al*., 1992). Rates for Indonesia and Vietnam were extracted from other published sources (Sarjadi, 1990; Pham *et al*., 1993), and the quality of these data is uncertain.

*Cancers with rates more similar to Australia born than country of birth rates*

Although oral cancer is one of the most common cancers in India, chiefly related to chewing tobacco (WHO, 1984), rates were not raised in migrants from India Sri Lanka. It was unclear whether the decreased rates were because these migrants, who are of high socioeconomic status (SES) and predominantly of mixed Anglo-Indian ethnicity, had never chewed tobacco, or whether they stopped chewing tobacco on coming to Australia. Mortality rates from oral cancer in Indian migrants to England and Wales are increased above rates of those born in England but these migrants are of lower SES than Indian migrants to Australia (Berra and Swerdlow, in preparation).

Stomach cancer is the most common cancer in most of East Asia (Parkin *et al*., 1993). However, in NSW it was significantly increased only in the China Taiwan born, and in none of the migrant groups was it the most common.

Rates of colorectal cancer were higher in migrants than in their country of origin, except in women born in the Philippines. However, in most migrants SIRs were significantly low in one or both sexes. The exceptions were the Hong Kong, Malaysia Singapore, and Indonesia born. Colorectal cancer risk in the Chinese in Singapore, China and the US has been associated with an increased food energy intake from fat (Whittemore *et al*., 1990), and an increased meat vegetable consumption ratio (Lee *et al*., 1989), and rates have been found to increase rapidly with transition to the American diet in Chinese migrants to the US (Yu *et al*., 1991). Rates in Singapore were lower in the ethnic Chinese born in China than in those born in Singapore (Lee *et al*., 1988). In Singapore during 1983–87 incidence rates of colorectal cancer in Indians were lower than rates in the Chinese (Parkin *et al*., 1992), reflecting the pattern seen in this study. A possible explanation is that Indians may be more likely to be vegetarian. At the 1991 Australian census, 18.8% of the India born, and 30.8% of the Sri Lanka born, compared with 3.6% of the Hong Kong born, classified themselves as Buddhist or Hindu (Bureau for Immigration Research, unpublished data), religions which encourage avoidance of meat.

Liver cancer tended to be much less common than in the countries of origin, except in the Vietnam and Indonesia born, but rates were generally still above those of the Australia born. The raised rates were consistent with the distribution of hepatitis B, the principal cause of this cancer in Asia (Anthony, 1984). Hepatitis B infection in Asians is usually acquired vertically or during early childhood (Anthony, 1984). If early infection were the sole risk factor, one would expect rates in migrants to be similar to country of birth rates. However, Tables IV and V show that, in those migrant groups with high country of origin rates, incidence rates tended to be lower in Australia. This is consistent with the action of co-factors, acting later in life, in the aetiology of liver cancer. Research in China suggests that one such factor may be aflatoxin ingestion (Yeh *et al*., 1989; Ross *et al*., 1992).

In males, low lung cancer rates in those born in Malaysia Singapore and India Sri Lanka were surprising given the almost equivalent rates of smoking in Asia- and Australia-born males found by the National Health Survey (Castles, 1992). Possible explanations for this pattern include differential smoking patterns among migrants from within the Asian region, differences in duration of smoking from the Australia-born population, and the high SES of these immigrant groups. No information on duration of smoking by country of birth was available.

In the immigrant groups of high SES, breast cancer rates were similar to those in the Australia born. In China Taiwan- and Vietnam-born women rates were low, but higher than in their countries of birth. In Singapore, rates of breast cancer were lower in ethnic Chinese women born in China than in those born in Singapore (Lee *et al*., 1988). Breast cancer is more common in women of higher SES (Petrakis *et al*., 1982) and in Chinese of high educational status in Singapore (Lee *et al*., 1993). Early age at first full-term pregnancy has been found to be protective against breast cancer in both Caucasians (Petrakis *et al*., 1982) and Chinese (Lee *et al*., 1993). The highest fertility rates in Australian immigrant women were in Filipino (3.2) and in China Taiwan- (2.5) and Vietnam-born (2.2) women (Castles, 1989), the three groups with the lowest rates of breast cancer. In addition, women born in Hong Kong, Malaysia Singapore and India Sri Lanka, who were more likely to delay child bearing until after the age of 25 (Castles, 1989), had higher rates of breast cancer. That obesity was more common in Australia-born than in Asia-born women (Castles, 1992) may also be related to the variations in breast cancer incidence.

Low SES is strongly associated with risk of cervical cancer (Christopherson and Nealon, 1981) and appeared to be a predictor of risk in this study. Rates were low in the high-SES India Sri Lanka born, despite high country of origin rates. Conversely, rates in Vietnam-born women, who were of low SES, were high, despite apparent low country of origin rates. However, recently published Vietnamese rates are from the north of Vietnam (Pham *et al*., 1993), which may have lower rates of cervical cancer than the south, where cervical cancer has been previously reported as constituting over 50% of all cancer in women (Parkin, 1986). Increased proportional incidence of cervical cancer has been described in Vietnamese migrants to Los Angeles County (Ross *et al*., 1991). Evidence points towards a sexually transmitted virus, the human papillomavirus (HPV), as the cause of the majority of cases of cervical cancer (Bosch *et al*., 1992). While we had no data on HPV infection, high levels of reactivity to tests for syphilis have been described in Vietnamese refugees in Australia (Bek and Levy, 1992).

Rates of prostate cancer were low in most immigrant groups, but were much higher than in the countries of origin. Rates were lowest in the China Taiwan born, who also have low mortality from prostate cancer in the US (King and Locke, 1980) and in Singapore (Lee *et al*., 1988). However, rates in the Hong Kong born, who were of high SES, were not significantly low. The difference in rates between the China Taiwan and Hong Kong born is compatible with environmental factors in the causation of this cancer, but could also potentially be explained by socio-economic differentials in usage of medical services. A high-fat diet has been implicated as a risk factor for prostate cancer (Greenwald, 1982). The similarity between patterns of colorectal cancer and prostate cancer in this study, with low rates in low SES migrants and Indian migrants and rates close to those of the Australia born in the Hong Kong born, is consistent with this hypothesis.

SIRs for testicular cancer were 50 or less in all immigrant groups. This is consistent with previous findings of low rates of testicular cancer in Taiwan (King and Locke, 1980), in Indian migrants to England and Wales (Berra and Swerdlow, in preparation) and in other Asian populations (Parkin *et al.*, 1992).

The non-significantly low rates of bladder cancer found in all Asian immigrant groups were in contrast to the high rates of bladder cancer previously described in male British, European and Middle Eastern migrants to NSW (McCredie *et al.*, 1990). It has been postulated that occupational exposure to hazardous chemicals in low-status jobs in Australia could explain these high rates. The absence of raised rates in Asian migrants may reflect the fact that they were the most recent migrants, and the potentially harmful exposures were no longer present, or that the carcinogenic effects of any Australian exposures were not yet apparent. In addition, the fact that many Asian migrants were of high SES would make them less likely to be occupationally exposed to hazardous chemicals.

*Cancers with rates more similar to country of birth than Australia-born rates*

Rates of nasopharyngeal cancer (NPC) were close to those in the countries of origin. Raised rates of NPC have been described in Chinese migrants in the US (King and Locke, 1980) and NSW (Zhang *et al.*, 1984; McCredie and Coates, 1989), and in Vietnamese migrants to the US (Ross *et al.*, 1991) and England and Wales (Swerdlow, 1991). In southern China, rates of NPC are thought to be higher in persons of low socioeconomic status (SES) (Yu *et al.*, 1986), but we found high rates in the Hong Kong born, who were of high SES. Population-based studies in southern China have found that consumption of Cantonese-style salted fish as a weaning food is a strong risk factor for NPC (Yu *et al.*, 1986, 1988). It might be expected then that rates would stay high in all Chinese who migrate after infancy. It has long been recognised that Epstein–Barr virus infection is associated with this cancer (de-Thé, 1993), and others have postulated genetic susceptibility as a strong risk factor (Ho *et al.*, 1982; Lu *et al.*, 1990). Our findings, of high rates in both the high-SES Hong Kong born and the lower SES China Taiwan born, could be explained either by genetic risk factors or by a risk factor acting early in life that was not differentially distributed by SES. The finding of raised SIRs in other immigrant groups was consistent with either genetic or cultural intermingling of the ethnic Chinese in South-East Asia.

Melanoma rates were consistent with the expected protective effect of skin pigmentation. Melanoma constituted about 8% of all registered cancers in the Australia born during the period of this review in NSW but accounted for less than 2% in most of the immigrant groups.

The high rates of lung cancer found in female migrants from China Taiwan, and in migrant groups with a high proportion of ethnic Chinese, were at odds with the low prevalence of smoking found in Asia-born immigrant women in Australia (Castles, 1992). However, high rates of lung cancer, and a high proportion of adenocarcinoma, which is less strongly associated with smoking than squamous cell carcinoma (Lam *et al.*, 1987; Morabia and Wynder, 1991), have been previously described in Chinese women in Singapore, Hawaii, Hong Kong, the US (MacLennan *et al.*, 1977; Gao *et al.*, 1988; Koo and Ho, 1990) and NSW (McCredie *et al.*, 1990). In China, it has been estimated that only 25–35% of lung cancer in females is attributable to tobacco smoking (Wu-Williams *et al.*, 1990; Liu *et al.*, 1992), other possible risk factors including a deficiency of vitamin A (McLennan *et al.*, 1977), passive smoking (Lam *et al.*, 1987) and indoor air pollution (Liu *et al.*, 1993).

For the majority of cancers, environment factors including change to an Australian environment as well as socioeconomic status of the migrant group, appeared to be the major influences on cancer incidence. Only for the most visible difference between the races, skin colour, was there evidence of a genetic trait which dominated cancer risk (melanoma). Rates of nasopharyngeal cancer, and of lung cancer in females, were also similar to country of birth rates, consistent with either early environmental or genetic risk factors.

**References**

ABS (AUSTRALIAN BUREAU OF STATISTICS). Cross-classified tables, age by birthplace by sex, census of the Commonwealth of Australia, 1971, 1976, 1981, 1986 (unpublished).

AUSTRALIAN BUREAU OF STATISTICS. (1993). Table BO8, 'Birthplace by sex, 1991'. In *Basic Community Profile*, Catalogue No. 2722.1. ABS: Canberra.

ANTHONY PP. (1984). Hepatocellular carcinoma: an overview. In *Virus Associated Cancer in Africa*, Williams O, O'Conor G, De-The G, and Johnson C. (eds) pp. 3–29. IARC. Scientific Publication No. 63. IARC: Lyon.

ARMSTRONG BK, WOODINGS TL, STENHOUSE NS AND MCCALL MG. (1983). *Mortality from Cancer in Migrants to Australia, 1962–71*. NH and MRC Research Unit in Epidemiology and Preventive Medicine, University of Western Australia: Perth.

BEK MD AND LEVY MH. (1992). *A Review of the New South Wales Refugee Medical Screening Program*. State Health Publication No. (EHSEB) 92-12. State Health Publications: Sydney.

BERRA A AND SWERDLOW AJ. Cancer incidence in migrants to England and Wales from the Indian subcontinent (in preparation).

BOROWSKI A AND SHU J. (1992). *Australia's Population Trends and Prospects 1991*. Australian Government Printing Service: Canberra.

BOSCH FX, MUNOZ N, SHAH KV AND MEHEUS A. (1992). Second International workshop on the epidemiology of cervical cancer and human papilloma virus. *Int. J. Cancer*, **52**, 171–173.

BUREAU OF IMMIGRATION RESEARCH. (1991). *Community Profiles: Australia Born, Malaysia and Brunei Born, Philippines Born, Vietnam Born*. Australian Government Printing Service: Canberra.

CASTLES I. (1989). *Overseas-born Australians, 1988*. Australian Bureau of Statistics, Commonwealth Government Printer: Canberra.

CASTLES I. (1992). *1989–90 National Health Survey, Health Survey, Health Risk Factors Australia*. Commonwealth Government Printer: Canberra.

CHEN J, CAMPBELL TC, LI J AND PETO R. (1990). *Diet, Lifestyle, and Mortality in China*. Oxford University Press: Oxford.

CHRISTOPHERSON WM AND NEALON NA. (1981). Uterine cancer: a comparative study of black and white women. In *Cancer among Black Populations*, Mettlin C and Murphy G (eds) pp. 185–195. Alan R Liss: New York.

COATES M AND MCCREDIE M. (1989). *Cancer in New South Wales Incidence and Mortality 1984*. New South Wales Cancer Registry: Sydney.

COTÉ RA. (1982). *Systematised Nomenclature of Medicine*. College of American Pathologists: Skokie IL.

DE THÉ G. (1993). Epidemiology, pathogenesis and prevention of EBV associated malignancies. In *The Epstein Barr Virus and Associated Diseases*. Tursz T, Pagano JS, Ablashi DV, de Thé G and Pearson GR (eds) pp. 15–30. Colloque INSERM/John Libbey Eurotext: Montrouge.

DOLL R. (1976). Comparison between registries. Age-standardised rates. In *Cancer Incidence in Five Continents, Vol. III*. Waterhouse J, Muir C, Correa P and Powell J (eds) IARC Scientific Publication No. 15 IARC: Lyon.

GAO YT, BLOT WJ, ZHENG W, FRAUMENI JF AND HSU CW. (1988). Lung cancer and smoking in Shanghai. *Int. J. Epidemiol.*, **17**, 277–280.

GREENWALD P. (1982). Prostate. In *Cancer Epidemiology and Prevention*. Schottenfeld D and Fraumeni Jr JF (eds) pp. 938–946. WB Saunders: Philadelphia.

GUNAWAN I. (1988) Indonesians. In *The Australian People*. Jupp J (ed.) pp. 550–551. Angus & Robertson: Sydney.

HO JHC, CHAN CL, LAU WH, AU GKH AND KOO LC. (1982). Cancer in Hong Kong: some epidemiological observations. *Natl Cancer Inst. Monogr.*, **62**, 47–55.

KELLY P. (1988). Settlement of Vietnamese refugees. In *The Australian People*. Jupp J (ed.) pp. 833–836. Angus & Robertson: Sydney.

KING H AND LOCKE FB. (1980). Cancer mortality among Chinese in the United States. *J. Natl Cancer Inst.*, **65**, 1141–1148.

KOO LC AND HO JHC. (1990). Worldwide epidemiological patterns of lung cancer in non-smokers. *Int. J. Epidemiol.*, **19** (Suppl. 1), S14–S23.

LAM TH, KUNG ITM, WONG CM, LAM WK, KLEEVENS JWL, SAW D, HSU C, SENVIRATNE S, LAM SY, LO KK AND CHAN WC. (1987). Smoking, passive smoking and histological types in lung cancer in Hong Kong Chinese women. *Br. J. Cancer*, **56**, 673–678.

LEE HP, DAY NE AND SHANMUGARATNAM K. (1988). *Trends in Cancer Incidence in Singapore 1968–1982*. IARC. Scientific Publications No. 91. IARC: Lyon.

LEE HP, GOURLEY L, DUFFY SW, ESTÈVE J, LEE J AND DAY NE. (1989). Colorectal cancer and diet in an Asian population – a case–control study among Singapore Chinese. *Int. J. Cancer*, **43**, 1007–1016.

LEE HP, GOURLEY L, DUFFY SW, ESTÈVE J, LEE J AND DAY NE. (1993). Risk factors for breast cancer by age and menopausal status: a case–control study in Singapore. *Cancer Causes Control*, **3**, 313–322.

LIU Z. (1992). Smoking and Lung cancer in China: combined analysis of eight case–control studies. *Int. J. Epidemiol.*, **21**, 197–201.

LIU Q, SASCO AJ, RIBOLI E AND HU MX. (1993). Indoor air pollution in Guangzhou, Peoples Republic of China. *Am. J. Epidemiol.*, **137**, 145–154.

LU S, DAY NE, DEGOS L, LEPAGE V, WANG PC, CHAN SH, SIMONS M, MCKNIGHT, B., EASTON D, ZENG Y AND DE-THÉ G. (1990). Linkage of a nasopharyngeal carcinoma susceptibility locus to the HLA region (letter). *Nature*, **346**, 470–471.

MACLENNAN R, DA COSTA J, DAY NE, LAW CH, NG YK AND SHANMUGARATNAM K. (1977). Risk factors for lung cancer in Singapore Chinese, a population with high female incidence rates. *Int. J. Cancer*, **20**, 854–860.

MCCREDIE M AND COATES MS. (1989). *Cancer Incidence in Migrants to New South Wales, 1972 to 1984*. NSW Cancer Registry: Sydney.

MCCREDIE M, COATES MS AND FORD JM. (1990). Cancer incidence in migrants to New South Wales. *Int. J. Cancer*, **46**, 228–232.

MCCREDIE M, COATES M, CHURCHES T AND TAYLOR R. (1991). Cancer incidence in New South Wales, Australia. *Eur. J. Cancer*, **27**, 928–931.

MCCREDIE M, COATES M, DUQUE-PORTUGAL F, SMITH D AND TAYLOR R. (1993). *Common Cancers in Migrants to NSW 1972–1990*. NSW Cancer Registry: Sydney.

MCMICHAEL AJ AND GILES GG. (1988). Cancer in migrants to Australia: extending the descriptive epidemiological data. *Cancer Res.*, **48**, 751–756.

MCMICHAEL AJ, BONETT A AND RODER D. (1989). Cancer incidence among migrant populations in South Australia. *Med. J. Aust.*, **150**, 417–420.

MOORE G. (1988). Anglo-Indians. In *The Australian People*. Jupp J (ed.) pp. 547–550. Angus & Robertson: Sydney.

MORABIA A AND WYNDER EL. (1991). Cigarette smoking and lung cancer cell types. *Cancer*, **68**, 2074–2078.

PARKIN DM (ed.) (1986). *Cancer Occurrence in Developing Countries*. IARC Scientific Publication No. 75. IARC: Lyon.

PARKIN DM, MUIR CS, WHELAN SL, GAO YT, FERLAY J AND POWELL J. (1992). *Cancer Incidence in Five Continents, Vol. VI*. IARC Scientific Publications No. 120. IARC: Lyon.

PARKIN DM, PISANI P AND FERLAY J. (1993). Estimates of the worldwide incidence of eighteen major cancers in 1985. *Int. J. Cancer*, **54**, 594–606.

PETRAKIS NL, ERNSTER VL AND KING MC. (1982). Breast. In *Cancer Epidemiology and Prevention*. Schottenfeld D and Fraumeni Jr JF (eds) pp. 855–870. WB Saunders: Philadelphia.

PHAM THA, PARKIN DM, NGUYEN TH AND NGUYEN BD. (1993). Cancer in the population of Hanoi, Vietnam, 1988–1990. *Br. J. Cancer*, **68**, 1236–1242.

PINNAWALA S. (1988). Sri Lankans. In *The Australian people*. Jupp J (ed.) pp. 805–808. Angus & Robertson: Sydney.

ROSS RK, BERNSTEIN L, HARTNETT NM AND BOONE JR. (1991). Cancer patterns among Vietnamese immigrants in Los Angeles County. *Br. J. Cancer*, **64**, 185–6.

ROSS RK, YUAN JM, YU MC, WOGAN GN, QIAN GS, TU JT, GROOPMAN JD, GAO YT AND HENDERSON BE. (1992). Urinary aflatoxin biomarkers and risk of hepatocellular carcinoma. *Lancet*, **339**, 943–946.

SARJADI. (1990). *Cancer incidence 1985–1989 in Semarang, Indonesia*. Diponegoro University Press: Semarang.

SWERDLOW AJ. (1991). Mortality and cancer incidence in Vietnamese refugees in England and Wales: a follow-up study. *Int. J. Epidemiol.*, **20**, 13–19.

WHITTEMORE AS, WU-WILLIAMS AH, LEE M, SHU Z, GALLAGHER RP, DENG-AO J, LUN Z, XIANGHUI W, KUN C, JUNG D, TEH CZ, CHENGDE L, YAO XJ, PAFFENBARGER RS AND HENDERSON BE. (1990). Diet, physical activity, and colorectal cancer among Chinese in North America and China. *J. Natl Cancer Inst.*, **82**, 915–926.

WORLD HEALTH ORGANIZATION (1984). Control of oral cancer in developing countries. *Bull. WHO*, **62**, 817–830.

WU-WILLIAMS AH, DAI XD, BLOT W, XU ZY, SUN XW, XIAO HP, STONE BJ, YU SF, FENG YP, ERSHOW AG, SUN J, FRAUMENI JF AND HENDERSON BE. (1990). Lung cancer among women in north-east China. *Br. J. Cancer*, **62**, 982–987.

YEH FS, YU MC, MO CC, LUO S, TONG MJ AND HENDERSON BE. (1989). Hepatitis B virus, aflatoxins, and hepatocellular carcinoma in Southern Guangxi, China. *Cancer Res.*, **49**, 2506–2509.

YU H, HARRIS RE, GAO YT, GAO R AND WYNDER EL. (1991). Comparative epidemiology of cancers of the colon, rectum, prostate and breast in Shanghai, China, versus the United States. *Int. J. Epidemiol.*, **20**, 76–81.

YU MC, HO JHC, LAI SH AND HENDERSON B. (1986). Cantonese-style salted fish as a cause of nasopharyngeal carcinoma: report of a case–control study in Hong Kong. *Cancer Res.*, **46**, 956–961.

YU MC, MO CC, CHONG WX, YEH FS AND HENDERSON B. (1988). Preserved foods and nasopharyngeal carcinoma: a case–control study in Guangxi, China. *Cancer Res.*, **48**, 1954–1959.

ZHANG YQ, MACLENNAN R AND BERRY G. (1984). Mortality of Chinese in New South Wales, 1969–1978. *Int. J. Epidemiol.*, **13**, 188–192.